# The association between alexithymia and internet addiction disorder in a large sample of Chinese undergraduates: the role of sexual assault experiences

**DOI:** 10.3389/fpubh.2025.1510630

**Published:** 2025-06-18

**Authors:** Fangxinrui Qiu, Wanjie Tang, Tao Hu, Xiong Lu, Siqi Wu, Xinyu Zhou

**Affiliations:** ^1^International Medical College, Chongqing Medical University, Chongqing, China; ^2^School of Public Health, West China Fourth Affiliated Hospital, Sichuan University, Chengdu, China; ^3^Department of Education and Psychology, Chengdu Normal University, Chengdu, China; ^4^Mental Health Centre, West China Hospital, Sichuan University, Chengdu, China; ^5^Department of Psychiatry, The First Affiliated Hospital of Chongqing Medical University, Chongqing, China; ^6^Key Laboratory of Major Brain Disease and Aging Research (Ministry of Education), Chongqing Medical University, Chongqing, China

**Keywords:** sexual assault experiences, alexithymia, internet addiction disorder, undergraduates, moderation

## Abstract

**Objective:**

While a positive association between alexithymia and Internet Addiction Disorder (IAD) has been established, previous studies are often limited by small sample sizes, lack of representative populations, and insufficient attention to intra-group differences, such as the impact of experiences like sexual assault.

**Method:**

Anonymous questionnaires assessing alexithymia and IAD were distributed to approximately 11,000 university students across six institutions in Southwest China, resulting in 7,890 valid responses. In addition to measures of alexithymia and IAD, data were also collected on experiences of sexual assault, psychological distress, and a range of sociodemographic variables.

**Results:**

Alexithymia was directly associated with IAD (*r* = 0.40), and past experiences of sexual assault were found to exacerbate the negative impact of alexithymia on IAD symptoms, even after controlling for psychological distress and gender. Individuals who had experienced sexual assault reported significantly higher levels of alexithymia, psychological distress, and IAD compared to those who had not.

**Conclusion:**

To address the high prevalence of IAD among college students, it is essential to screen for difficulties in emotional identification and expression, and to provide support for improving these skills. Targeted interventions are especially important for vulnerable groups, such as survivors of sexual assault, to help reduce the risk of IAD. Future longitudinal studies are needed to further explore these relationships.

## 1 Introduction

Internet Addiction Disorder (IAD) was first mentioned as a potential mental disorder in the Diagnostic and Statistical Manual of Mental Disorders, Fifth Edition (DSM-5), reflecting that more evidence was needed before it could be formally classified as a mental disorder. IAD is characterized by an individual’s inability to control their internet use, including impulsive online behavior and, in severe cases, the inability to maintain a normal life (1). This disorder predominantly affects adolescents (2) and young adults (3), significantly impairing cognitive and emotional functioning, as well as overall quality of life (4).

Furthermore, IAD has been associated with mental illness (2) and challenges in societal adaptation (5). A meta-analysis conducted by Pan et al. (6) estimated the global prevalence of IAD to be around 7%. However, research in China by Shao et al. (7) revealed a higher prevalence among college students, approximately 11%, surpassing the global average. Thus, in-depth research into the prevalence of IAD and its underlying psychosocial mechanisms among young people could provide crucial insights for understanding its extensive effects and inform the development of effective prevention and control measures.

### 1.1 Psychosocial mechanisms associated with Internet Addiction Disorder (IAD)

Several psychosocial mechanisms have been linked to Internet Addiction Disorder (IAD), with alexithymia being identified as a potential pathogenic factor, though further studies are needed to confirm this relationship (8). Alexithymia is characterized by an inability to identify, analyze, or express one’s own emotions, as well as a difficulty in recognizing emotions in others (9). This condition is also associated with an externally oriented thinking style (10). According to Emotional Processing Theory, individuals with alexithymia may engage in compulsive behaviors, such as excessive internet use, as a way to avoid experiencing feelings of inner emptiness (9). Due to their lack of emotional awareness, people with alexithymia often have limited inner experiences, show minimal interest in dreams, and exhibit a tendency toward concrete thinking and externalized living styles.

This lack of internal emotional life often leads alexithymic individuals to struggle with forming relationships, gravitate toward external thinking patterns, or indulge in compulsive internet use as a means of establishing external communication or coping mechanisms (11). A scoping review by Mahapatra and Sharma (8) found a significant positive association between alexithymia scores and the severity of IAD. However, much of the research on this relationship has relied on convenience samples, many of which included fewer than 500 participants, and reported correlation coefficients varying widely from 0.2 to 0.52. Therefore, larger and more representative samples are needed to confirm the positive correlation between alexithymia and IAD severity.

### 1.2 The relationship between sexual assault, alexithymia, and IAD

While a positive relationship between alexithymia and IAD is suggested, the dynamics of this relationship may vary under different circumstances, such as in cases of sexual assault. Sexual assault encompasses vaginal, oral, and anal penetration, and is defined more broadly than the legal definition of rape, which typically refers to nonconsensual penile penetration (12, 13), can have long-term psychological impacts, leading to distressing emotions and risky behaviors (14–16). Research has shown that victims of sexual assault are more likely to be alexithymic compared to non-victims (17). Additionally, sexual assault has been linked to higher rates of problem drinking, drug use (18), and IAD among adolescents and young adults (19). This suggests a potential relationship between sexual assault experiences, alexithymia, and IAD.

Dworkin et al. (20) reviewed global data and found that the incidence of sexual assault in community surveys varied widely, ranging from 0 to 59%, likely reflecting differences in sample selection. In the United States, one study reported that 26.6% of 17-year-old females and 5.1% of males had experienced sexual abuse or assault (21). Although data on sexual assault rates within Chinese communities are limited, a recent meta-analysis estimated that approximately 9% of Chinese adolescents have experienced some form of sexual abuse (22). Therefore, it is likely that a certain proportion of university students have experienced sexual assault, which may influence the relationship between alexithymia and internet addiction in this population. A previous study by Hahn et al. (23) found that a history of sexual assault could moderate the relationship between emotional dysregulation and alcohol use. Since traumatic experiences have also been closely linked to both IAD and alexithymia (24), it is plausible that sexual assault experiences may exacerbate the pathway from alexithymia to IAD.

Given the evidence, alexithymia appears to be closely related to IAD, with past experiences of sexual assault potentially acting as moderating factors. This study, therefore, aims to explore the relationships between alexithymia and IAD in a large, representative sample, as well as the possible moderating roles of sexual assault history. The following three hypotheses were developed for this study, with demographic variables such as the experience of being left-behind children (LBC) and psychological distress being used as control variables. In China, LBC are those raised by grandparents while their parents work in cities, often with limited parental contact, which is related to alexithymia (25) and IAD (26). Additionally, psychological distress are also considered to be related to IAD (27) and alexithymia (28), which can also be treated as a control variable. Moreover, gender factors are also an important influence on IAD (29, 30) which need to be controlled.

Our hypothesis is as follows:

*H1*: Alexithymia is positively and directly related to IAD.

*H2*: Sexual assault victims are more likely to have both alexithymia and IAD.

*H3*: Sexual assault experiences moderate the relationship between alexithymia and IAD.

## 2 Methods

### 2.1 Participants

**Figure 1:**
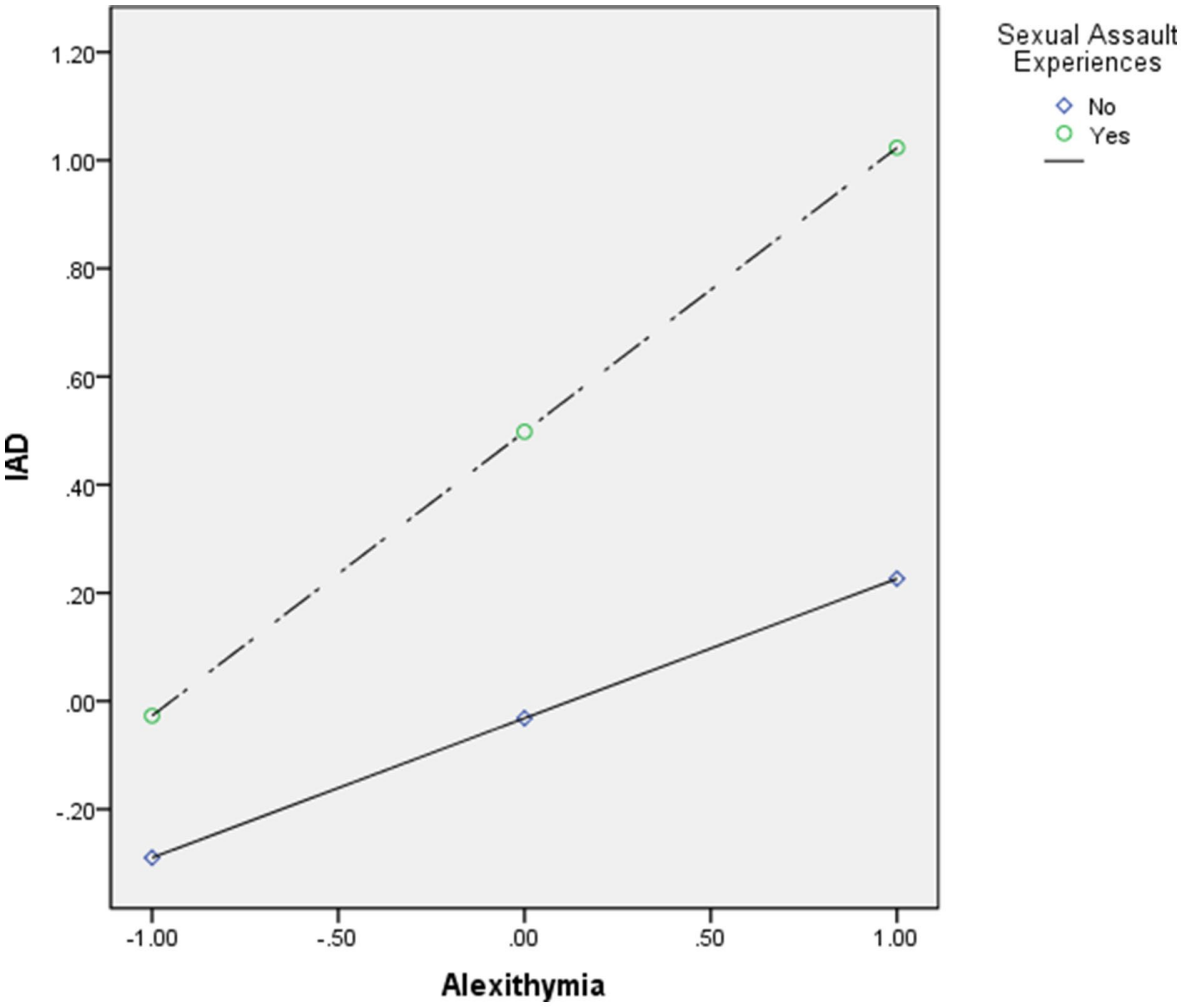
Moderating effect of sexual assault experiences on the relationship between alexithymia and Internet Addiction Disorder (IAD) after controlling for psychological distress and sex.

Out of the 11,017 undergraduate students initially contacted from six universities in Chongqing Municipality and Sichuan Province, China, 7,890 agreed to participate and completed the questionnaire, resulting in a response rate of 71.7%. After excluding 227 questionnaires that did not meet the quality criteria, data from 7,663 valid responses were analyzed. The participants ranged in age from 15 to 28 years, with a mean age of 18.3 years (SD = 0.81). The majority of the respondents (6,898) were of Han ethnicity, while 765 belonged to various ethnic minority groups (Figure 1).

### 2.2 Procedure

The study was conducted across six universities—Sichuan University, Chengdu University, Chengdu Normal University, Chengdu University of Technology, Chongqing Jiaotong University, and Yangtze Normal University—located in Chongqing and Chengdu. Ethical approval was obtained from the Ethics Committee of Sichuan Psychological Society. The survey link will be sent to the student affairs department of each school, which will be responsible for distributing it to the designated counselors. The counselors will then share it with the students through WeChat groups, and the login password will be set to each student’s ID number, and each student ID can only be used once. A total of 11,017 students were contacted online via WeChat Groups. The invitation included a brief description of this study, an informed consent form, and a link to the formal questionnaire. Participants who consented to participate signed the informed consent form and followed the link to complete the questionnaire. All questions were set as mandatory, ensuring that all submitted questionnaires were complete. The responses were collected over a two-week period. The questionnaire used a matrix survey format and included items to ensure the quality of responses (For example, we included general knowledge questions, such as “What is the capital of China?” to check attention).

### 2.3 Measures

#### 2.3.1 Toronto Alexithymia Scale-20 (TAS-20)

The Toronto Alexithymia Scale-20 (TAS-20) was utilized to assess alexithymia among participants. This scale has been widely validated across different cultures, including Chinese (31), and demonstrates strong reliability, as well as good convergent, construct, and discriminant validity (32, 33). The TAS-20 consists of three subscales: difficulty identifying feelings, difficulty describing feelings, and externally oriented thinking. Each item is rated on a five-point scale ranging from 1 (strongly disagree) to 5 (strongly agree). The total score is the sum of the 20 item scores, yielding a range from 20 to 100. A score of 61 or higher indicates severe alexithymia. In the current study, the Cronbach’s *α* coefficient for the TAS-20 was 0.85.

#### 2.3.2 Young’s Internet Addiction Test (IAT)

Young’s revised 20-item Internet Addiction Test (IAT) was employed to measure Internet Addiction Disorder (IAD) over the past month. A sample item is, “Do you find yourself spending more time online using smartphones or computers than you originally intended?” The Chinese version of the IAT has been shown to have high reliability and validity in college student populations (34, 35). The scale includes 20 items, each rated on a 5-point Likert scale ranging from 1 (almost never) to 5 (almost always). The total score ranges from 20 to 100, with scores of 50 or more indicating IAD and scores of 80 or more indicating severe IAD. The Cronbach’s *α* for the IAT in this study was 0.92.

#### 2.3.3 Kessler-6 scale

The Kessler-6 (K6) is a brief, widely used self-report questionnaire designed to measure nonspecific psychological distress, particularly symptoms of anxiety and depression, in the general population. Developed by Kessler et al. (36), it serves as a screening tool to identify individuals at risk of non-specific psychological distress and is validated for use in both clinical and community settings (36). This scale consists of 6 items assessing emotional states over the past 30 days (e.g., feeling nervous, hopeless, restless, worthless, or that everything is an effort). Each item is rated on a 5-point Likert scale (0 = “None of the time” to 4 = “All of the time”). Total scores range from 0 to 24, with higher scores indicating greater psychological distress. The cut-off score is set at 13; a score of 13 or higher indicates severe psychological distress. In the current study, the Cronbach’s *α* for the K6 was 0.73.

#### 2.3.4 Sexual assault experiences

Sexual assault (including vaginal, oral, and anal penetration) was assessed using adapted questions from previous studies (37, 38). Participants were asked, “Have you ever been sexual assulted?” The response options were dichotomous (yes or no), in line with previous research (37, 39).

#### 2.3.5 Sociodemographic variables

The demographic information collected included age, gender, and other potentially influential sociodemographic factors such as ethnic affiliation, relationship status, left-behind status, and only-child status.

### 2.4 Statistical analysis

All statistical analyses were performed using SPSS version 22. Initially, descriptive statistics were calculated for the variables. We will conduct a normality test on the distribution of three variables including psychological distress, IAD, and alexithymia between individuals with and without a history of sexual assault. If the distribution is normal, we will perform a *t*-test; if it is not normal, we will use a non-parametric test for comparison. Pearson’s correlation analysis was used to explore the relationships between the main variables. The moderating effects of sexual assault history on the relationship between alexithymia and IAD controlled for psychological distress and socio-demographic variables were assessed using Model 1 of Hayes’s PROCESS macro for SPSS (40).

## 3 Results

### 3.1 Demographics and clinical characteristics

Among the 7,663 participants, 3,837 were female (50.1%), 4,713 were only children (61.5%), 1,017 reported having romantic partners (13.3%), and 911 had left-behind experiences (11.9%). A total of 281 participants had experienced sexual assault (see Table 1). The prevalence of IAD was higher among individuals with alexithymia compared to those without (27.4% vs. 7.0%, *p* < 0.001). Additionally, participants who had been sexually assaulted exhibited a higher rate of IAD compared to those who had not been assaulted (44.1% vs. 8.5%, *p* < 0.001). The LBC showed no significant difference in IAD, with *χ*^2^ = 3.27, *p* = 0.08. The proportion of female experiencing sexual assault is higher, with a chi-square value of 8.76 and a *p*-value of 0.003. The rate of IAD is higher among males, with a chi-square value of 12.26 and a *p*-value less than 0.001.

**Table 1:** Multidimensional assessment of socio-demographic determinants and subclinical symptomatology among undergraduates (*N* = 7,663).

Variables	*N*	%
Gender		
Male	3,826	49.9
Female	3,837	50.1
Age		
15–17	905	11.8
18–19	6,340	82.7
20 or above	418	5.5
Only-child status		
Yes	4,713	61.5
No	2,950	38.5
Marriage		
Single	6,646	86.7
In a relationship or married	1,017	13.3
Ethnicity		
Han	6,898	90
National minority	765	10
Left-behind experience		
Yes	911	11.9
No	6,752	88.1
Being sexual assaulted		
Yes	281	3.7
No	7,382	96.3
Alexithymic		
Yes	638	8.3
No	7,025	91.7
Severe psychological distress		
Yes	102	1.7
No	7,561	98.7
Internet Addiction Disorder (IAD)		
Yes	669	8.7
No	6,994	91.3

### 3.2 Correlations between major variables

Pearson’s correlation results are also calculated. A moderate positive correlation was observed between alexithymia and IAD (*r* = 0.40, *p* < 0.001). Furthermore, positive correlations were found between psychological distress and both alexithymia (*r* = 0.47, *p* < 0.001) and IAD (*r* = 0.36, *p* < 0.001).

### 3.3 Non-parametric comparison of internalizing and externalizing problems between individuals with and without sexual assault experiences

Table 2 presents the results of a non-parametric comparison of internalizing and externalizing problems between individuals with and without sexual assault experiences. The findings indicate significant differences across three variables:

**Table 2:** Non-parametric comparison of internalizing and externalizing problems between individuals with and without sexual assault experiences (*N* = 7,663).

History of sexual assault	Internalizing/externalizing problems	*Z*	*p*	Effective value (r)
	Alexithymia, M (P_25_, P_75_)	−16.24	<0.001	0.19
No	43 (37–51)			
Yes	61 (46.5–68)			
	IAD, M (P_25_, P_75_)	−14.11	<0.001	0.16
No	30 (24–38)			
Yes	45 (31.5–60)			
	PD, M (P_25_, P_75_)	−9.79	<0.001	0.11
No	2 (1–5)			
Yes	5 (2–8)			

IAD, internet addiction disorder; PD, psychological distress.

Alexithymia: Individuals with sexual assault experiences reported higher levels (*M* = 61, P_25_ = 46.5, P_75_ = 68) compared to those without (*M* = 43, P_25_ = 37, P_75_ = 51), with a *Z* value of −16.24 and a *p*-value < 0.001, resulting in an effect size (r) of 0.19.

IAD: Those with sexual assault experiences also exhibited greater IAD (*M* = 45, P_25_ = 31.5, P_75_ = 60) than those without (*M* = 30, P_25_ = 24, P_75_ = 38), with a *Z* value of −14.11 and a *p*-value < 0.001, yielding an effect size (r) of 0.16.

Psychological distress: Psychological distress was higher in individuals with sexual assault experiences (*M* = 5, P_25_ = 2, P_75_ = 8) compared to those without (*M* = 2, P_25_ = 1, P_75_ = 5), with a *Z* value of −9.79 and a *p*-value < 0.001, resulting in an effect size (r) of 0.11.

Overall, these results highlight significant internalizing and externalizing problems associated with sexual assault experiences (see Table 2).

### 3.4 The moderating role of sexual assault experiences on the relationship between alexithymia and IAD when controlled for co-variables

Table 3 illustrates the role of sexual assault experiences as a moderator in the relationship between alexithymia and IAD, while controlling for psychological distress and gender.

**Table 3:** Sexual assault experiences (M, moderator) as a moderator for alexithymia (X, independent variable) on Internet Addiction Disorder (IAD) (Y, outcome variable) after controlling for psychological distress and sex (*N* = 7,663).

Predictors	Model1 (IAD) (Y)				Model2 (IAD) (Y)			
	*β*	SD	*t*	95% CI	*β*	SD	*t*	95% CI
Alexithymia (X)	0.27	0.024	14.40^***^	0.29-0.69	0.26	0.012	22.02^***^	0.24 ~ 0.28
Sexual assault (M)					0.53	0.074	7.12^***^	0.38 ~ 0.68
Alexithymia (X) × Sexual Assault (M)					0.27	0.045	6.00^***^	0.18 ~ 0.35
Confounding variable: PD					0.21	0.011	18.70^***^	0.19 ~ 0.23
Confounding variable: Sex (male)					0.25	0.020	12.14^***^	0.20 ~ 0.28
*R* ^2^			0.07				0.24	
*F*			207.32^***^				477.90^***^	

***p < 0.001. IAD, internet addiction disorder; PD, psychological distress.

Alexithymia: In both models, alexithymia significantly predicts IAD, with coefficients of *β* = 0.27 (Model 1) and *β* = 0.26 (Model 2), both highly significant (*p* < 0.001).

Sexual assault: In Model 2, sexual assault experiences is also a significant predictor of IAD (*β* = 0.53, *p* < 0.001).

Interaction effect: The interaction between alexithymia and sexual assault experiences is significant (*β* = 0.27, *p* < 0.001), indicating that the relationship between alexithymia and IAD is stronger for individuals with sexual assault experiences.

Psychological distress: This confounding variable is also a significant predictor of IAD (*β* = 0.21, *p* < 0.001).

Male: In the present study, male is also a significant predictor of IAD (*β* = 0.25, *p* < 0.001).

Overall, the results suggest that sexual assault experiences moderate the impact of alexithymia on IAD, highlighting the importance of considering sexual trauma history in understanding these relationships. The models show a substantial increase in explanatory power, with *R*^2^values of 0.07 in Model 1 and 0.24 in Model 2.

## 4 Discussion

The study revealed significant associations between alexithymia, sexual assault experiences, and IAD, with alexithymia showing a strong positive correlation to IAD. Participants with sexual assault experiences exhibited markedly higher rates of IAD, alexithymia, and psychological distress compared to non-assaulted individuals. Sexual assault experiences moderated the alexithymia-IAD relationship, intensifying this link, while psychological distress independently linked to IAD. These findings underscore the critical role of sexual trauma history in shaping vulnerability to maladaptive coping behaviors like IAD.

As hypothesized, our findings support Emotional Processing Theory, which suggests that individuals with alexithymia may engage in compulsive behaviors, such as excessive internet use, to avoid confronting feelings of inner emptiness (9). This is consistent with previous researches (41–43). According to alexithymia processing theory, alexithymics seek stimuli to compensate for the loss of affective input and tend to over-control themselves to offset their lack of awareness (44). These compensation mechanisms may contribute to addictive behaviors such as IAD (45). Neurobiological research has also confirmed that there is a connection between alexithymia and the neural mechanisms of reward and loss processing (46, 47). Glucksman’s theory (48) also suggests that alexithymia is related to emotional dysfunction, where developmental disturbances result in self-representation deficits such as inner blankness and emptiness, and a lack of affective experiences and expressions. To combat this inner emptiness, alexithymic individuals may resort to addictive behaviors like IAD (49). The difficulties in emotional identification, expression, and communication experienced by alexithymics may drive them to excessive internet use to fulfill social needs (50) and to compensate for emotional deficiencies (44), potentially leading to avoidance of interpersonal communication (51). Thus, our study provides robust evidence supporting the need for further exploration of the relationship between alexithymia and IAD, offering valuable insights into the psychological etiology of IAD.

Another significant finding is that a history of sexual assault moderated the relationship between alexithymia and IAD. This study is the first to confirm that past sexual assault experiences exacerbate the adverse effects of alexithymia on IAD symptoms. Past research has also shown that trauma can lead to some addiction problems (52). This finding extends previous research by identifying factors that intensify the relationship between alexithymia and youth IAD. One possible explanation is that the emotional dysregulation associated with alexithymia (53, 54) may be intensified by past sexual assault experiences. Sexual assault survivors often have heightened interpersonal sensitivities and may be more affected by stressful events (55, 56). They may engage in self-denial and emotional suppression as defense mechanisms (55, 57), which aligns with the emotional expression difficulties of alexithymics. Consequently, individuals with alexithymia who have experienced sexual assault may seek escape from their harsh reality by turning to the internet for social support and emotional comfort (8, 58). Additionally, when support systems fail, survivors may find a voice through social media that they cannot express in real-world interactions (59). This area warrants further exploration to deepen understanding of the moderating effects of sexual assault on the alexithymia-IAD relationship.

Our research also found that psychological distress and being male are influencing factors for IAD. Mental health issues have long been considered risk factors for IAD, and they are interrelated (60). Our study confirms this, suggesting that psychological problems may lead to IAD as a maladaptive way to regulate emotions (35). Since our study is cross-sectional, it is also possible that IAD impacts mental health issues. Additionally, being male is a risk factor for IAD, which further confirms that males are more likely than females to experience IAD (61, 62). This may also indicate that excessive internet use serves as a means for males to regulate and improve their emotions or cope with stress (30).

### 4.1 Conclusions and implications

This study, utilizing a large representative sample, explored the relationship between alexithymia and IAD and assessed the impact of intra-group differences such as sexual assault experiences on this relationship. The study highlights the complexity of IAD and its psychosocial etiology, particularly in relation to alexithymia. Future research should focus on interventions that improve emotional identification and expression among IAD sufferers and investigate the mechanisms linking IAD and alexithymia. Larger studies are needed to further elucidate these mechanisms.

Given that characteristics such as a history of sexual assault influence IAD differently, interventions should be tailored to address these specific characteristics. Addressing difficulties in emotional recognition and expression can mitigate IAD susceptibility and improve treatment outcomes.

### 4.2 Limitations

Despite the robust study design and significant findings, several limitations must be acknowledged. Firstly, the study’s sample, while large and representative, was drawn from China. Cultural factors may influence the applicability of these results to other countries, necessitating caution when generalizing findings. Secondly, the reliance on self-report scales introduces potential subjective bias. Future research should incorporate objective measurement methods to validate these findings. Thirdly, use a single question to measure sexual assault is a limitation, as the severity, duration and other classifications of sexual assault are not clearly defined. Fourthly, in Chinese culture, many individuals who have experienced sexual assault often feel a sense of shame, which may lead to a reluctance to disclose their experiences, even in anonymous surveys. As a result, the reported prevalence of sexual assault may be underestimated, and this should be taken into consideration. Finally, the retrospective nature of the study limits causal inference. Longitudinal studies are needed to better understand the sequence and causality of the observed relationships.

## Data Availability

The original contributions presented in the study are included in the article/supplementary material, further inquiries can be directed to the corresponding authors.
